# Predicting Risk of Severe Toxicity and Early Death in Older Adult Patients Treated with Chemotherapy

**DOI:** 10.3390/cancers15184670

**Published:** 2023-09-21

**Authors:** Jaime Feliu, Ana Belén Custodio, Alvaro Pinto-Marín, Oliver Higuera, Miriam Pérez-González, Laura del Pino, Leticia Ruiz-Jiménez, Darío Sánchez-Cabero, Isabel Viera, Ana Jurado, Enrique Espinosa

**Affiliations:** 1 Oncology Department, Hospital La Paz Institute for Health Research—IdiPAZ, Hospital Universitario La Paz, 28029 Madrid, Spain; anabcustodio@gmail.com (A.B.C.); alvaropintomarin@gmail.com (A.P.-M.); oliverhiguera@gmail.com (O.H.); mperezgonzalez2@salud.madrid.org (M.P.-G.); lauradpq@gmail.com (L.d.P.); letiruizgimenez@hotmail.com (L.R.-J.); dario.sc88@gmail.com (D.S.-C.); isavieraher@gmail.com (I.V.); ana.jurado@salud.madrid.org (A.J.); eespinosa00@hotmail.com (E.E.); 2 Cátedra UAM-AMGEN de Oncología Médica y Medicina Paliativa, Facultad de Medicina, Universidad Autónoma de Madrid, 28029 Madrid, Spain; 3 Centro de Investigación Biomédica en Red del Cáncer (CIBERONC), Instituto de Salud Carlos III, 28029 Madrid, Spain

**Keywords:** cancer, older adult patient, chemotherapy, toxicity, prognostic, early death

## Abstract

**Simple Summary:**

Cancer therapy in the older adult remains a challenge; much is unknown about treatment tolerance and impact on survival in this age group. Factors predicting serious toxicity and early death (ED) were identified in a series of 234 older adult patients treated with chemotherapy. Two simple and reliable scores based on these factors were developed to predict the risk of grade 3–5 toxicity and ED, which can help in treatment planning and the implementation of corrective measures.

**Abstract:**

Background: Determining the risk of grade 3–5 toxicity and early death (ED) is important to plan chemotherapy in older adult patients with cancer. Our objective was to identify factors predicting these complications at the time of treatment initiation. Methods: 234 patients aged ≥70 were subjected to a geriatric assessment and variables related to the tumor and the treatment were also collected. Logistic regression multivariable analysis was used to relate these factors with the appearance of grade 3–5 toxicity and ED. Predictive scores for both toxicity and ED were then developed. Results: Factors related to grade 3–5 toxicity were hemoglobin, MAX2 index, ADL, and the CONUT score. Factors related to ED were tumor stage and the GNRI score. Two predictive scores were developed using these variables. ROC curves for the prediction of toxicity and ED were 0.71 (95% CI: 0.64–0.78) and 0.73 (95% CI: 0.68–0.79), respectively. Conclusions: Two simple and reliable scores were developed to predict grade 3–5 toxicity and ED in older adult patients with cancer. This may be helpful in treatment planning.

## 1. Introduction

The incidence of cancer increases with age, so that approximately 50% of patients are ≥70 years-old at the time of diagnosis [1]. Older adults patients are very heterogenous regarding health status, physical performance, comorbidities, and frailty. The identification of factors related to tolerance to therapy and prognosis is important to make therapeutic decisions [2].

Pharmacodynamic and pharmacokinetic factors, as well as tissue tolerance change with aging, which increase the risk of toxicity [3]. The risk of developing severe toxicity (grade 3–5) in older adults patients receiving chemotherapy for advanced cancer has been reported to be as high as 30–50% [4,5]. On the other hand, the prognosis of this population not only depends on tumor stage and performance status, as in other age groups, but also a number of different conditions, mainly comorbidities [2]. For these reasons, cancer therapy in the older adult remains a challenge.

Geriatric assessment (GA) can identify older patients at higher risk of toxicity and predict their survival, which helps optimize therapy [6]. Two recent large randomized clinical trials (Geriatric Assessment-Driven Intervention (GAIN) [7] and Geriatric Assessment for Patients 70 Years and Older (GAP70+)) [8] demonstrated their utility to reduce serious chemotherapy-related toxic effects in older adults with cancer. However, it is not clear that all components of GA can reliably predict these outcomes. For instance, comorbidity [9], performance status [10], nutritional status [11], social support [12], and cognitive status [13] seem to better predict for toxicity. Several toxicity risks scores have been developed [5,12,14]. The most widely used are the CARG (Cancer and Aging Research Group) score and the CRASH (Chemotherapy Risk Assessment Scale for High-Age Patients) score. The former has 11 variables related with age, therapy, cancer type, geriatric assessment, hemoglobin, and renal function) [12]. The latter correlates the risk of hematological toxicity with IADLs, diastolic blood pressure, LDH levels, and the chemotherapy scheme used (MAX2 score). The non-hematological toxicity was related to ECOG PS, cognitive status, the presence of malnutrition and the type of chemotherapy (MAX2score) [14]. Both scores have been validated in some studies [15,16] but not in others [4,17]. Likewise, scores predicting prognosis have been proposed that are based on performance status and nutritional status [2,18,19,20].

Poor nutrition can be detected in 15–73% of patients with cancer [21], with the percentage depending on the type of cancer, disease stage, and age. Cancer can decrease food intake, cause metabolic disorders, and increase basal energy consumption. The tumor itself releases cytokines and hormones that contribute to the syndrome [22]. Poor nutrition increases mortality, reduces quality of life and physical performance, and lowers tolerance to drugs, including chemotherapy [21,23,24].

Few studies have studied the relation between nutritional status and events such as chemotherapy toxicity [10,24] or early death in the older adult (ED), defined as death within the first 6 months from the diagnosis of cancer [19,20]. Up to 83% of older patients with cancer have poor nutritional status, most commonly in digestive tumors (28–75%) and in metastatic disease (64–76%). Some markers of immune nutrition have been described: the Geriatric Nutritional Risk Index (GNRI) [25], the Prognostic Nutritional Index (PNI) [26], and the Controlling Nutritional Status (CONUT) [27]. Their utility has not been studied in this context.

The identification of patients at increased risk of toxicity or ED is relevant. It can lead to the initiation of antineoplastic therapy or, alternatively, prioritization of palliative care. Our objective in the present study was to assess the utility of GA and nutritional markers to predict grade 3–5 toxicity and ED in older adults patients receiving chemotherapy.

## 2. Materials and Methods

This prospective, single-center study included 234 patients between February 2014 and June 2018. All patients had been treated in the service of oncology of Hospital Universitario La Paz.

Inclusion criteria were as follows: (1) Confirmation of solid cancer—any stage—via biopsy or cytology; (2) Age ≥ 70; (3) Eastern Cooperative Oncology Group performance status (PS) (ECOG-PS) 0–2; (4) Initiation of chemotherapy either adjuvant or in first line; (5) Able to read Spanish (questionnaires of GA were in Spanish).

Patients receiving immunotherapy or targeted therapies were excluded unless they were combined with chemotherapy.

All patients signed informed consent. The study was approved by the local ethical committee (13 June 2013; IRB number: 1349).

### 2.1. Study Scheme

Standard tumor staging was performed depending on tumor type. GA (Supplementary Table S1) was completed before the initiation of therapy. Questionnaires were supplied by an investigation nurse. The nurse completed the following: comorbidities (Cumulative Illness Rating Scale Geriatrics—CIRS-G and Charlson index) [28,29], ECOG PS [30], body mass index (BMI), frailty (short physical performance battery—SPPB, comprising the 4 m gait speed, standing balance, and five-repetition chair-stand test) [31,32], cognitive status (Short Portable Mental Status Questionnaire, Pfeiffer’s test) [33], and percentage of weight loss in the last 6 months. Patients themselves reported on functional status (basic activities in daily living—ADL) [34], instrumental activities in daily living (IADL) using the Lawton Index [35], number of falls in the last six months, medications, nutrition, psychological state [36], social support and function [37,38], ability to take medications unassisted, and the Vulnerable Elders Survey-13 (VES-13) [39]. Patients requiring assistance to complete questionnaires were helped by a member of the investigation team. The following clinical variables were collected: age, sex, education, home status, marital status, hearing, type of cancer and stage, and parameters from the blood test before the initiation of therapy (hemoglobin, white blood cell count, platelets, liver function test, albumin, cholesterol, basal creatinine, and creatinine clearance) [40].

Cachexia was defined as weight loss >5% or weight loss greater than 2% in patients with BMI < 20 kg/m^2^ [41]. Muscle mass loss was not available, so we did not include it in the definition of cancer cachexia. The next parameters of nutrition were determined: (1) GNRI was calculated using serum albumin concentration and body weight as described elsewhere [24]; (2) PNI was calculated with the lymphocyte count of the peripheral blood and serum albumin as described by Ontera et al. [25]; (3) The CONUT score was based on the levels of cholesterol, albumin, and lymphocytes [26]. GNRI was calculated using the formula: GNRI = 14.89 × serum albumin (g/dL) + 41.7 × [present body weight (kg)/ideal body weight (kg)]. The ideal weight was defined as [height (m)] 2 × 22. PNI was calculated as follows: 10 × albumin (g/dL) + 0.005 × lymphocytes count (/mm^3^). The CONUT score is the sum of the following: albumin concentration (≥3.5 mg/dL: 0 points, 3.0–3.49 mg/dL: 2 points, 2.5–2.99 mg/dL: 4 points, and <2.5 mg/dL: 6 points), plus total lymphocyte count (≥1600/mm^3^: 0 points, 1200–1599/mm^3^: 1 point, 800–1199/mm^3^: 2 points, and <800/mm^3^: 3 points), plus cholesterol level (≥180 mg/dL: 0 point, 140–179/mm^3^: 1 point, 100–139/mm^3^: 2 points, and <100/mm^3^: 3 points).

The cause of death was collected from the hospital data base and the national death registry.

The MAX2 index was used to assess the risk of toxicity for every scheme of chemotherapy [42]. It is the average of the highest frequency of both grade 4 hematologic and grades 3–4 non-hematologic toxicities. It is reproducible across cancer types and studies and is sensitive to differences among chemotherapy regimens. In addition, the CARG Toxicity Score was calculated in all patients [12].

Decisions regarding chemotherapy regimen and doses were made by the treating oncologists, who were blinded to the results of GA. Chemotherapy intensity for the first cycle of treatment was categorized as standard or reduced therapy per European Society for Medical Oncology (ESMO) guidelines [43] or the National Comprehensive Cancer Network (NCCN) [44]. When ESMO and NCCN guidelines disagreed about treatment recommendation, the less intensive option was considered as the reference or standard. Reduced therapy was defined as follows: (1) Single-agent chemotherapy or combination at reduced doses (<85% of the dose of chemotherapy reference standard) [45]; (2) Single-agent chemotherapy at standard dose when combination chemotherapy was the first option according to guidelines.

All patients were followed until the end of chemotherapy or death. Deaths occurring within 6 months of treatment initiation were considered as early death (ED). The treating oncologists collected toxicity before every cycle and at the end of treatment. The Common Terminology Criteria for Adverse Events (CTCAE) v. 4.03 was used to grade toxicity [46].

### 2.2. Statistical Analysis

Descriptive analysis was performed for basal patients’ characteristics. The X^2^ test was used to correlate categorical variables with grade 3–5 toxicity and ED, whereas the independent *t* test was used for continuous variables. Correlation between categorical variables was assessed using the Spearman rho test. Multicolineality between variables was define by a rho test value ≥ 0.50. The Pearson correlation analysis was used for continuous variables.

Factors that could contribute to grade 3–5 toxicity and ED were selected for logistic regression analysis. Variables significant at the 5% level in univariate analysis and did not show multicollinearity were included in the Cox multivariate forward stepwise logistic regression analysis. Odds ratios (ORs) were reported with their 95% CIs. *p* < 0.05 was considered statistically significant for all comparisons. The Youden index was used to determine the optimal cut-point for continuous variables. Categorical variables were dichotomized according to clinically relevant cut offs.

The amount of accounted variance was determined using the Nagelkerke correlation coefficient [R2]. Model calibration and discrimination were assessed via the Hosmer–Lameshow test and the area under the receiver operating characteristic (ROC) curve [47,48]. For the development of the score, each factor was assigned a particular score based on its b coefficient. The b coefficient for each risk factor was divided by the lowest b coefficient and rounded to the nearest whole number [49,50]. The risk score was then applied to each patient. We divided the sample in three risk strata (low, medium, and high risk of grade 3–4 toxicity and ED) based on approximate tertiles of risk score. The Chi-square testing was used to compare the risk groups. We used the bootstrap method (1000 repetitions) for internal validation of the risk score. Bootstrap validation is a method of random resampling from a given set of samples to simulate the effect of drawing samples from the same population.

Analyses were carried out by using SPSS software (version 26; SPSS, Chicago, IL, USA).

## 3. Results

### 3.1. Patients’ Characteristics

A total of 241 patients had a basal GA. Two of them withdrew consent, two died before the initiation of therapy, two moved to another center after the first course of treatment, and one received targeted therapy without chemotherapy, so the series eventually included 234 patients.

Table 1 shows basal clinical characteristics, including demographic data, results of GA, type of chemotherapy, and laboratory blood tests. The median age was 78 (range 70–92), 35% of patients being ≥80. Sixty-two percent were male. Regarding the type of tumor, 57% were digestive (colorectal 86%, gastric 10%, and esophageal cancer 4%), 12% pulmonary, and 10% genitourinary. Forty-five percent of patients had stage I–III disease and 55% stage IV. ECOG-PS was ≤1 in 91% of patients.

Combination chemotherapy was administered in 54% of cases. Standard doses were used in 65% of patients, more commonly in those aged 70–79 (81% vs. 44%; *p* < 0.0001). Five percent of patients received primary prophylaxis with colony-stimulating factors.

#### 3.1.1. Geriatric Assessment

Two or more comorbidities were present in 31% of patients (Charlson index). Disability was detected via ADL and IADL in 18% and 47% of patients, respectively. Fifteen percent had at least one fall in the last 6 months and 21% had a SPPB score ≤ 6. Unintentional weight loss ≥5% appeared in 31%, being ≥10% in 10% of patients. The median BMI was 25.8 (range 17–44), with 11% of patients having <20. One hundred eighty-four patients (33%) had criteria of tumor cachexia. The GNRI score ranged between 77 and 116, (median 99.6), the PNI score between 37 and 80 (median 40.4), and the CONUT score between 0 and 8 (median 1.3). Nine percent of patients had ≥3 errors in the Pfeiffer test, which suggests cognitive impairment. When education and cultural level were considered, cognitive impairment was confirmed in 8%. A VES-13 score ≥3 suggesting frailty was present in 62% of patients, being more common in those over the age of 80 (52% vs. 77%; *p* < 0.0001).

#### 3.1.2. Toxicity of Chemotherapy and Early Death

After a median follow-up of 6.9 months (minimum follow-up 6 months), data on toxicity and survival were available for 234 patients. Grade 3–5 toxicity appeared in 32% of patients: hematological in 12% and non-hematological in 30% (some patients had both).

The most common grade 3–5 hematological side effects were neutropenia (8%) and anemia (3%). The most common grade 3–5 non-hematological side effects were diarrhea (13%), fatigue (10%), and neuropathy (5%). There was one toxic death due to febrile neutropenia and sepsis. Dose reduction was applied in 28% of patients. Ten percent of patients had early discontinuation due to toxicity.

Twenty-two percent of patients died within 6 months after the initiation of therapy. ED occurred in 9% of patients with early-stage disease and 32% of those with advanced disease. Causes of ED in the former were disease progression (40%), intercurrent conditions (20%), treatment toxicity (20%), worsening of previous conditions (10%), and unknown causes (10%); in the latter, disease progression (60%), intercurrent conditions (15%) comorbidity (12%), toxicity (9%), and unknown causes (4%) occurred.

#### 3.1.3. Variables Predicting Grade 3–5 Toxicity and Early Death

The univariate analysis included parameters from the geriatric assessment, as well as clinical and laboratory parameters (Table 2). Factors related to grade 3–5 toxicity were ADL ≤ 5, CONUT score ≥ 1, hemoglobin < 12.5 g/dL, and MAX2 index ≥ 0.45 (Table 2).

Factors related to ED were stage IV disease, hemoglobin < 12.5 g/dL, cachexia, PNI score ≤ 41, CONUT score ≥ 1, GNRI score ≤ 98, creatinine clearance < 60 mL/min, albumin ≤ 3.5 g/dL, GGT > 125 UI/L, alkaline phosphatase > 150 UI/L, ADL ≤ 5, and IADL ≤ 7 (Table 2).

In the multivariate analysis, factors related to grade 3–5 toxicity were hemoglobin, MAX2 index, ADL, and CONUT score (Table 3); factors related to ED were stage and GNRI score (Table 4).

#### 3.1.4. Predictive Models for Grade 3–5 Toxicity and Early Death

**Figure 1:**
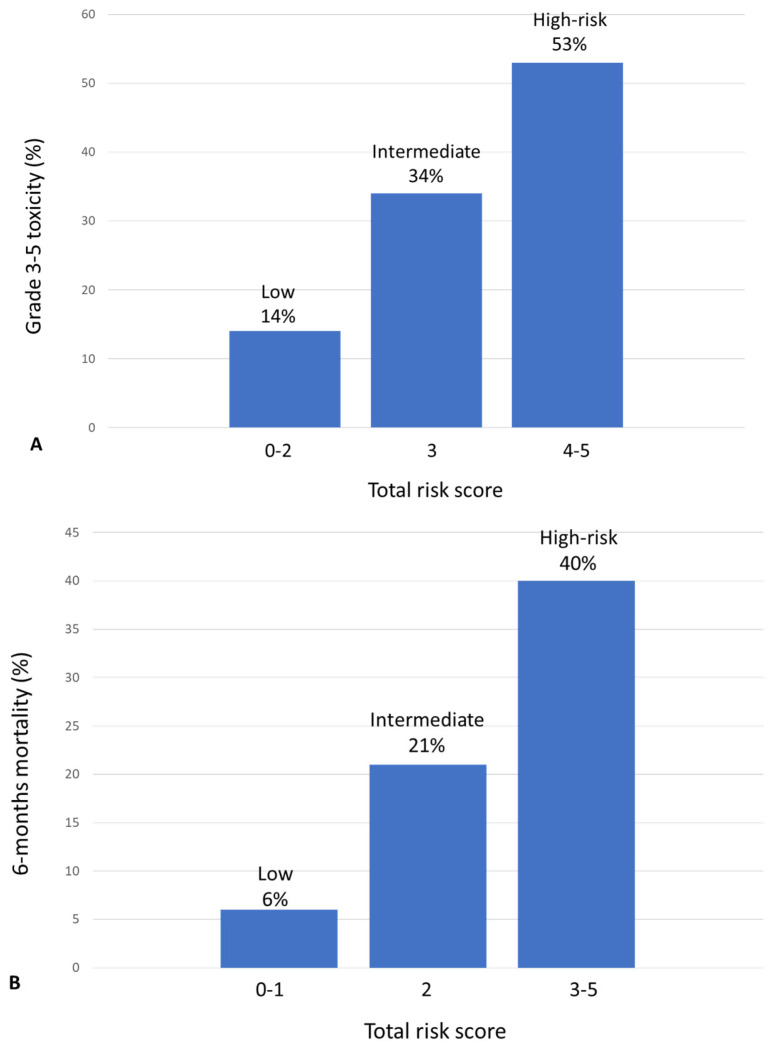
Ability of different risk scores to predict grade 3–5 toxicity (**A**) and 6-month mortality (**B**).

Each variable identified in the multivariate analysis was given a value based on its b coefficient. These values were used to create a predictive score. The predictive score was then applied to each patient, so that patients were classified into three groups according to their risk to develop toxicity: low risk (0–2 points, grade 3–5 toxicity in 14% of them), intermediate risk (3 points grade 3–5 toxicity in 34%), and high risk (4–5 points, grade 3–5 toxicity in 53%) (Figure 1). The proportion of patients in each group was 35%, 34%, and 31%, respectively. Differences in the rates of 3–5 toxicity were statistically significant (*p* < 0.001). The area under the receiver operation characteristics (ROC) curve was 0.71 (95% CI: 0.64–0.78) (Supplementary Figure S1).

The predictive value of the CARG Toxicity Score was also assessed. The median value was 7. Risk of developing grade 3–5 toxicity was 18% for the low-risk group (0–5 points), 34% for the intermediate group (6–10 points), and 28% for the high-risk group (≥10 points). The area under the ROC curve was 0.58 (95% CI 0.51–0.64).

A predictive score for ED was developed. The risk of ED was 6% for the low-risk group (0–1 points), 21% for the intermediate group (2 points), and 40% for the high-risk group (3–5 points). The proportion of patients in each group was 40%, 30%, and 30%, respectively. Differences in the rates of ED were statistically significant (*p* < 0.000). The area under the ROC curve was 0.73 (95% CI: 0.68–0.79) (Supplementary Figure S1).

Both predictive models were calibrated using the Hosmer–Lemeshow goodness of fit test. *p* values were 0.56 for toxicity (95% CI, 0.50–0.62) and 0.58 (95% CI, 0.53–0.63) for ED, suggesting good calibration.

## 4. Discussion

The risk of serious toxicity and ED should be evaluated in older patients who are going to initiate chemotherapy, because some of them may be better served with less aggressive options or even just supportive care. Our results suggest that scores based on nutritional parameters can provide useful information. We found that the following variables were related to the risk of having grade 3–5 toxicity: ADL score, hemoglobin, CONUT score, and the MAX2 index. Variables predicting ED were tumor stage and the GNRI score.

In our series, the rate of malnutrition ranged from 31% if a threshold of 6-month weight loss was ≥5% was considered, and 47% with a threshold of PNI score ≤45. These numbers are within what has been reported for older adults patients with cancer [23].

Malnutrition not only relates with toxicity from chemotherapy and ED, as found in our series, but also with long-term mortality, comorbidity, prolonged hospitalization, frequent readmission, decreased quality of life, and increased health expenditure [51,52].

Weight loss, BMI and albumin levels have been correlated with toxicity from chemotherapy [11]. An increased risk has also been described in older patients with increased resting energy expenditure, a factor that could be more predictive than the CARG score and the CRASH score [53]. In our series, weight loss, albumin level and BMI did not correlate with the risk of toxicity, but nutrition as assessed by the CONUT score remained significant in the multivariate analysis. The CONUT score is based on the levels of albumin, lymphocytes, and cholesterol, so it provides information about nutritional status and inflammation. Serum albumin levels decrease by 15–20% with ageing, further descents seen in cancer patients with malnutrition and inflammation [54]. This may elevate the plasma-free fraction of drugs such as cisplatin, methotrexate, the taxanes, and etoposide [55] and increase their toxicity. The lymphocyte count relates to the immune response, the cholesterol level with nutritional status, and the immune response [56]. Other variables included in our score predicting toxicity were the MAX2 index, hemoglobin, and the ADL score. The MAX2 index quantifies the aggressiveness of chemotherapy, so its correlation with the risk of toxicity makes sense. There is no clear explanation for the role of hemoglobin, although it reflects the bone marrow capacity. Also, the chronic inflammation seen in advanced cancer inhibits hematopoiesis [57]. Other studies have identified anemia as a risk factor for toxicity and, in fact, this variable is included in the CARG score [15]. Regarding ADL, impaired functional status has been shown to increase the risk of toxicity or the need to reduce the doses of chemotherapy [13,14], although they have been usually related to IADL score. However, there are also studies that have linked the ADL score with the risk of toxicity or the need to reduce the doses of chemotherapy [10,58,59].

We and others [4,17,60] could not validate the CARG score. This score clearly identified a low-risk group for toxicity but did not find differences between the intermediate- and high-risk groups. Our series of patients differed from those in the study by Hurria et al. [12], which may explain the discrepancy: older age, higher proportion of male patients, higher proportion of gastrointestinal tumors, lower proportion of patients with severe weight loss, lower percentage of patients receiving standard dose treatment, cultural and racial differences. In fact, some authors suggest that the validation of the CARG score would require a population with similar characteristics [61].

Twenty-two percent of our patients died in the first 6 months after the initiation of chemotherapy. The availability of simple and reliable tools to predict this could have an impact in treatment planning and resource management. More specifically, therapeutic decisions could be adapted to life expectancy and patients’ preferences in the older adult. In the case of early-stage disease, this could lead to forgoing adjuvant therapy.

Soubeyran et al. reported that factors related with ED in older patients starting chemotherapy were advanced disease, a low MNA score, male sex, and poor mobility [2]. Use in daily practice is difficult as they did not develop a nomogram or prognostic score. Some tools have been developed to predict ED in this population (at 100 days [19] and at 6 months [18,20]). These tools include five to six variables, for instance disease stage, nutritional status, and performance status [18,19,20], and some of these—such as ECOG-PS—can be subjected to interpretation. Our prognostic score includes only two variables, tumor stage and the GNRI score. The latter is easily calculated from routine laboratory parameters. So, this score is simple and objective. The already-mentioned tools were developed in patients receiving either chemotherapy or just palliative care [19,20], whereas our study included only patients planned to begin chemotherapy, making it more useful to oncologists seeing such patients.

The GNRI score was initially used to assess the nutritional status of older patients with chronic diseases [25]. Further studies demonstrated that the GNRI score could assess prognosis in non-geriatric diseases such as sepsis, chronic obstructive pulmonary disease, hemodialysis, and heart failure [62,63,64,65]. The GNRI score may identify patients at risk for developing postoperative complications and, in patients different tumor types (lung, kidney, pancreas, esophageal-gastric, and lymphoma), it may predict short- and long-term survival [66]. Our study is the first to assess its utility in predicting ED in older patients treated with chemotherapy. GNRI provides information about nutritional status and systemic inflammation. Inflammation decreases the efficacy of chemotherapy and impairs survival [67]. The prognostic value of markers of inflammation—such as GNRI, CONUT, or PNI has been demonstrated in patients with cancer [68]; although, in our series, GNRI proved superior to CONUT and PNI to predict ED. Of those variables included in our score for ED, GNRI is the only one that can be modified, so patients with high-risk GNRI could benefit from some kind of pre-habilitation to improve their nutritional status, performance status, and, eventually, survival.

The main strengths of our study are as follows: (1) The scores focus on two relevant issues to plan treatment in older patients with cancer; (2) The scores are based on variables that are cheap and easy to obtain in clinical practice; (3) The scores are simple and easy to calculate. Limitations are as follows: (1) This is a single-institution study, so a multicentric and prospective study should be performed to validate the scores; (2) Hematological tumors were excluded and some tumor types were underrepresented: many patients with breast, prostate, or lung tumors receive hormonal therapy, immunotherapy, or targeted therapies and the scores should be validated in these cases; (3) Our patients do not represent the whole population of older people with cancer, as only those amenable to receive chemotherapy were selected. In fact, our patients had a good performance status, few falls, and few comorbid conditions. For this reason, we do not know if the scores would keep their predictive value in patients with serious comorbid conditions, organ failure, or poor performance status, i.e., situations with a contraindication for chemotherapy.

## 5. Conclusions

A combination of factors related to geriatric assessment, nutritional status, laboratory tests, tumor stage and treatment can predict grade 3–5 toxicity from chemotherapy and ED in older patients with cancer. Two scores were built that predict both situations. These tools can be useful to plan therapy and lead to the implementation of corrective measures to reduce the risk or avoid such complications. Validation in further studies is warranted.

## Figures and Tables

**Table 1:** Patients’ characteristics.

Characteristics	Total (n = 234)
Age, median (SD) years	78 (5.1)
Sex	
Male	144 (62%)
Female	90 (38%)
Type of cancer	
Gastrointestinal	134 (57%)
Lung	28 (12%)
Genitourinary	24 (10%)
Breast	14 (6%)
Other	34 (15%)
Metastatic status	
M0	106 (45%)
M1	128 (55%)
Chemotherapy	
Standard therapy	153 (65%)
Reduced therapy or monotherapy	81 (35%)
MAX2 Index	
0	74 (32%)
1	122 (52%)
2	38 (16%)
Granulocyte colony-stimulating factors	
Yes	11 (5%)
No	223 (95%)
ECOG PS	
0	61 (26%)
1	152 (65%)
2	21 (9%)
Charlson comorbid index	
0	104 (44%)
1	58 (25%)
≥2	72 (31%)
ADL	
6	190 (81%)
≤5	44 (18%)
IADL	
8	124 (53%)
≤7	110 (47%)
SPPB	
>7	185 (79%)
≤6	49 (21%)
No. falls in the last 6 months	
None	199 (85%)
≥1	35 (15%)
Pfeiffer test	
0–2 errors	213 (91%)
≥3	21 (9%)
VES 13	
0–2	88 (38%)
≥3	146 (62%)
Unintentional weight loss %	
< 5%	161 (69%)
≥5%	73 (31%)
Body Mass Index	
<20	26 (11%)
20–30	174 (75%)
>30	34 (14%)
Cachexia	
Yes	78 (34%)
No	156 (66%)
GNRI score	
≤98	108 (46%)
>98	126 (54%)
PNI score	
≤45	110 (47%)
>45	124 (53%)
CONUT score	
0	88 (38%)
>1	146 (62%)
Toxicity	
Grade 0–2	159 (68%)
Grade 3–5	75 (32%)
Early death (<6 months from diagnosis)	
Yes	52 (22%)
No	182 (78%)

Abbreviations: ECOG PS, Eastern Cooperative Oncology Group performance status; ADL, activities of daily living; IADL: Instrumental activities of daily living; SPPB, Short Physical Performance Battery VES-13, Vulnerable Elders Survey-13; GNRI: Geriatric Nutritional Risk Index; PNI, Prognostic Nutritional Index; CONUT, Controlling Nutritional Status.

**Table 2:** Factors associated with toxicity and early death.

Variable	Toxicity			Early Death		
	No	Yes	*p* Value	No	Yes	*p* Value
ECOG PS						
2	12	9	0.266	13	8	0.067
0–1	147	66		169	44	
ADL						
≤5	24	20	**0.034**	28	16	**0.012**
6	135	55		154	36	
IADL						
≤7	72	38	0.441	77	33	**0.006**
8	87	37		105	19	
VES-13						
≥3	94	52	0.132	108	38	0.071
0–2	65	23		74	14	
Cachexia						
Yes	50	28	0.372	52	26	**0.008**
No	109	47		120	26	
GNRI score						
≤98	71	37	0.502	71	37	**0.000**
>98	88	38		111	15	
PNI score						
≤41	68	42	0.058	72	38	**0.0000**
>41	91	33		110	14	
CONUT score						
>1	90	56	**0.007**	106	40	**0.014**
0	69	19		76	12	
Creatinine Clearance mL/min						
<60	60	37	0.093	68	29	**0.017**
≥60	99	38		114	23	
Hemoglobin g/dL						
<12.5	60	47	**0.000**	74	33	**0.004**
≥12.5	99	28		108	19	
Albumin g/dL						
≤35	28	18	0.251	23	23	**0.000**
>35	131	57		158	29	
MAX2 index						
≥0.45	100	60	**0.009**	126	34	0.599
0–0.44	59	15		56	18	
Metastatic status						
M1	87	41	0.994	86	42	**0.000**
M0	72	37		96	10	
GGT (IU/L)						
>125	30	17	0.498	29	18	**0.003**
≤125	129	58		153	34	
Alkaline Phosphatase (IU/L)						
>150	30	18	0.364	31	17	**0.014**
≤150	129	57		151	35	

Abbreviations: ECOG PS, Eastern Cooperative Oncology Group performance status; ADL, activities of daily living; IADL: instrumental activities of daily living; VES-13, Vulnerable Elders Survey-13; GNRI: Geriatric Nutritional Risk Index; PNI, Prognostic Nutritional Index; CONUT, Controlling Nutritional Status; GGT, gamma glutamil transferase; IU, international unit. Significant values in bold

**Table 3:** Variables related to grade 3–5 toxicity in multivariate analysis.

Variable	β	SE	*p* *	OR (95% CI)	Score
CONUT score ≥ 1	0.711	0.335	0.034	2.036 (1.056–3.926)	1
MAX2 index > 0.45	1.145	0.373	0.002	3.143 (1.514–6.525)	1
Hemoglobin ≤ 12.5 g/dL	1.109	0.373	0.000	3.033(1.631–5.639)	1
ADL ≤ 5	1.171	0.398	0.003	3.225 (1.477–7.045)	1

SE = standard error; CI = confidence interval; OR = odds ratio; * *p* values were calculated using a two-sided Wald test for multivariable analyses. ADL, activities of daily living. CONUT, Controlling Nutritional Status.

**Table 4:** Variables related to early death in multivariate analysis.

Variable	β	SE	*p* *	OR (95% CI)	Score
Stage IV	1.412	0.402	0.000	4.102 (1.866–9.019)	1
GNRI score	1.028	0.215	0.000	2.795 (1.833–4.263)	1

SE = standard error; CI = confidence interval; OR = odds ratio; * *p* values were calculated using a two-sided Wald test for multivariable analyses. GNRI: Geriatric Nutritional Risk Index (0 point > 98; 1 point 92–98; 2 point 82–92; 3 point ≤ 81).

## Data Availability

De-identified individual data might be made available following publication by reasonable request to the corresponding author.

## Supplementary Materials

The following supporting information can be downloaded at: https://www.mdpi.com/article/10.3390/cancers15184670/s1, Table S1: Summary of comprehensive geriatric assessment domains and elements. Figure S1: Receiver operating characteristic (ROC) analyses to assess the capacity of the predicting grade 3–4 toxicity (**A**) and death at 6 months (**B**).
