# Supplementary material for: Predicting Risk of Severe Toxicity and Early Death in Older Adult Patients Treated with Chemotherapy
Source: Cancers (Basel). 2023 Sep 21;15(18):4670. doi: 10.3390/cancers15184670 (PMC10526243; doi:10.3390/cancers15184670)
Supplement: Supplementary file 1 [file cancers-15-04670-s001.zip › cancers-2539298-supplementary.pdf]

Table S1. Summary of Comprehensive Geriatric Assessment Domains and Elements

Domain	Elements of Assessment
Functional status	ECOG performance status [30]<br>Activities of daily living [34]<br>Instrumental activities of daily living [35]<br>Physical performance test SPPB [31]<br>Nº of falls in the last 6 months
Comorbidity	Cumulative Illness Rating Scale for Geriatrics (CIRS-G score) [28]<br>Charlson index [29]
Psychological status	Hospital Anxiety Scale [36]
Cognitive status	Pfeiffer test [32]
Social support	MOS Social Support Survey [37]
Nutritional status	Body mass index<br>Percent unintentional weight lost in the last 6 months<br>GNRI score [25]<br>PNI score [26]<br>CONUT score [27]

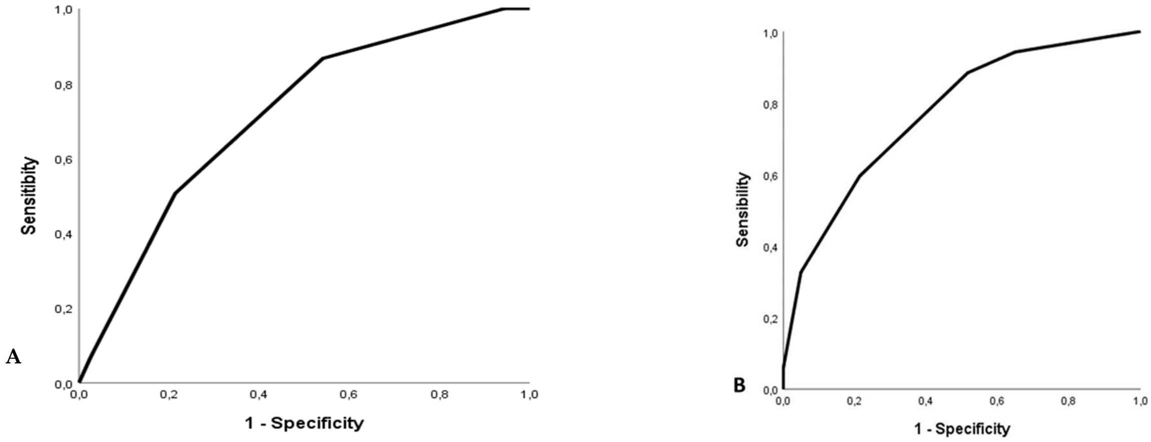

Figure S1. Receiver operating characteristic (ROC) analyses to assess the capacity of the predicting grade 3-4 toxicity (A) and death at 6 months (B).
